# Tabulae Anatomical Osteologiae

**Published:** 1886-02

**Authors:** 


TABULAE ANATOMICAL OSTEOLOGIAE.—*By Carolo H. von Klein, A. M., M. D., Cincinnati, Ohio.*—This useful Anatomical Hand-Atlas is presented in Latin and at a trifle as to cost, in order, as the author states in his preface, that the work may reach every physician, surgeon, dentist and medical student in the civilized world. The merits of this Anatomical Atlas are such as to commend it to all interested in the study of anatomy, and it cannot fail to be of the greatest service to the student of anatomy on account of the admirable manner in which it is arranged and the finely executed engravings by which it is illustrated. Anatomical works of recent years may be said never to grow old, but the difference in the style and arrangement of such treatises commend some over others as more comprehensive text-books. We consider the work of Dr. Klein to be a great improvement over many others of a like subject.

